# HLA-C, DRB1 and DQB1 alleles involved in genetic predisposition to psoriasis vulgaris in the Slovak population

**DOI:** 10.1007/s12223-012-0213-7

**Published:** 2012-11-27

**Authors:** Ivana Shawkatová, Juraj Javor, Zuzana Párnická, Peter Kozub, Mária Žilínková, Peter Frey, Stanislav Ferenčík, Milan Buc

**Affiliations:** 1 Department of Immunology, Comenius University School of Medicine, Nám. odborárov 14, 811 08 Bratislava, Slovakia; 2 Department of Dermatovenereology, University Hospital, Mickiewiczova 13, 813 69 Bratislava, Slovakia; 3 Dermatology Clinic, Slovak Medical University and University Hospital, Ružinovská 6, 826 06 Bratislava, Slovakia; 4 Outpatient Dermatovenereology Clinic CUTIS Ltd, Nábrežná 20, 940 01 Nové Zámky, Slovakia; 5 Institute of Immunology, University Hospital Essen, Hufelandstr. 55, 45147 Essen, Germany

## Abstract

Psoriasis vulgaris is a complex chronic skin disease with immunological and genetic background. The most important predisposing genetic factors in psoriasis are genes of the human leukocyte antigen (HLA) region. Accumulative evidence has shown that several HLA alleles are closely associated with psoriasis; however, they tend to vary in different racial and ethnic backgrounds. One hundred forty-seven unrelated Slovak patients with psoriasis vulgaris (average age at onset 28 ± 14 years) were genotyped for the HLA-C, DQB1 and DRB1 alleles by the polymerase chain reaction using sequence-specific primers. Allele frequencies observed in the group of psoriatic patients were compared to those obtained in the ethnically matched control group comprising 194 subjects with no history of psoriasis. Susceptibility to psoriasis vulgaris in our study group is significantly associated with HLA-C*06 (odds ratio (OR) = 3.85), DRB1*07 (OR = 2.56) and DQB1*02 (OR = 1.09), respectively, whereas DRB*01 (OR = 0.05) is associated negatively. Hereby, we provide the first report on the association of HLA-C, DRB1 and DQB1 alleles with psoriasis in the Slovak population. Our findings confirm HLA-C*06 and DRB1*07 as the most important genetic risk factors for psoriasis. However, the role of HLA genes as causative in the pathogenesis of the disease remains unclear. Identification of genetic factors that increase the risk of psoriasis is a precondition that helps to elucidate the pathogenesis of this troubling disease and identify targets for a more specific and effective therapy.

## Introduction

Psoriasis vulgaris (PV) is a common chronic skin disease with a worldwide distribution, affecting 1–3 % of the general population (Henseler 1998; Kurd and Gelfand 2009). Like many other immune-mediated diseases, psoriasis results from a combined action of multiple genes and environmental triggers. The age at disease onset is highly variable; however, studies have shown that 75–90 % of patients develop psoriasis before the age of 40, with a peak around puberty and a smaller peak around 50–60 years of age (Henseler and Christophers 1985). Among psoriatic patients, up to 40 % develop psoriatic arthritis (PsA)—an associated inflammation of the joints (Weger 2010).

The human leukocyte antigen (HLA) region on chromosome 6p21 is well known to carry the most important genetic factors in the susceptibility to psoriasis, whereas the critical segment appears to be a 300-kb interval at the centromeric end of HLA class I, named PSORS1 (Nair et al. 2000; Martinez-Borra et al. 2003). The first reports on the association of both HLA class I and class II antigens (e.g. HLA-B13, B17, DR7) with psoriasis were obtained by serologic methods already in the 1970s (Russel et al. 1972; Brenner et al. 1978). In the following years, molecular genotyping was introduced, and many independent groups have proven that among HLA alleles, HLA-C*06 confers the major and most consistently demonstrated risk factor (Elder et al. 1994; Szczerkowska-Dobosz 2005; Pasic et al. 2009). HLA-C*06 carriers have a significantly higher risk of developing PV with risk ratios ranging in Caucasoid psoriatic patients from 3 to 36 (Cassia et al. 2007). However, not all HLA-C*06 psoriasis-associated haplotypes appear to confer an equal risk of PV in different populations as reviewed by Elder et al. (2001). Multiple HLA alleles have been identified in increased occurrence rates, most probably because of linkage disequilibrium in the HLA region. In the German, Italian and Thai population, the extended haplotype (EH) HLA-C*06-B57-DRB1*0701-DQA1*0201-DQB1*0303, named according to the B allele EH-57.1, was reported to be highly overrepresented in patients with psoriasis (Schmitt-Egenolf 1996; Choonhakarn et al. 2002). Another similar predisposing haplotype, HLA-C*0602-B57-DRB1*0701-DQA1*0201-DQB1*0201 (EH-57.2), was observed in Croatian patients (Kastelan et al. 2003). However, many of the associated alleles tend to vary among patients of different racial and ethnic backgrounds as reviewed by Cassia et al. (2007).

The aim of our study was to provide the first survey of the HLA-C as well as HLA-DRB1* and DQB1* allele distribution in Slovak psoriatic patients as a contribution to the precise definition of HLA class I- and class II-associated loci in different ethnic populations.

## Materials and methods

### Study group

One hundred forty-seven unrelated patients with chronic stable plaque PV were included in the study. They were recruited at random via several outpatient dermatovenereology clinics in Slovakia. Detailed clinical parameters of the study group were recorded by the dermatologists, and they are summarized in Table 1. Mean age at disease onset was 28 years (range 7–67, standard deviation (SD) ± 14). Of the patients, 82.3 % had disease onset before 40 years of age and 17.6 % after 40. Severity and extent of the disease were estimated using the scoring system Psoriasis Area and Severity Index (PASI). The average PASI score was 22 ± 17.

**Table 1:** Characteristics of the study group

Parameter	Psoriatic subjects (*n* = 147)
Gender ratio M/F	95/52
Mean age ± SD (years)	47 ± 12
Mean onset age ± SD (years)	28 ± 14
Onset at ≤40/>40 years	121/26
Familiar occurrence +/−	67/80
Psoriatic arthritis +/−	68/79
Average PASI score ± SD	22 ± 17

*SD* standard deviation, *PASI* Psoriasis Area and Severity Index

According to the criteria proposed by Henseler and Christophers (1985), patients may be subdivided into two subsets: type I psoriasis (age at onset before 40, presence of a positive family history and strong association with HLA-C*06) and type II (age at onset after 40, no family history and weak association with HLA-C*06). At first, we have subdivided our patients into subsets according to the following criteria: age of onset ≤40 versus >40 years and/or a positive family history of psoriasis versus negative history. Family history was considered positive when at least one first- or second-degree relative had psoriasis. No statistically significant differences were found when comparing the HLA allele frequencies observed in those subsets (data not shown).

Similarly, we have divided our patients into a subset with psoriatic arthritis (46.3 % of the psoriatic patients) and a subset without signs of PsA (remaining 53.7 % of subjects). Again, no significant differences in frequencies were observed between the PsA-positive versus PsA-negative subsets. Therefore, we decided to report our results on the whole group of our psoriatic patients regardless of the above-mentioned criteria (Table 1).

### Population controls

The reference group comprised 194 unrelated subjects without a history of PV who were randomly recruited from blood and bone marrow donors. Data on the control subjects have been reported elsewhere (Cechova et al. 1998; Kulcsarova et al. 2000). All patients and controls were Caucasoids of Slovak descent. Slovakia is a Central European country with ethnically homogenous population. Romanies who constitute a fraction of about 5 % were not included in this study.

A written informed consent for enrolling in the study and for personal data management was obtained from all patients and control subjects. All investigations were carried out in accordance with the principles of the Declaration of Helsinki.

### HLA typing

Both HLA class I and class II alleles were determined by molecular typing that proved superior to serological methods due to higher specificity and sensitivity (Bunce et al. 1996). DNA was extracted from whole blood using a modified salting-out procedure (Miller et al. 1988). Genotyping was performed using the PCR-SSP technique according to the 12th Workshop protocol using Olerup SSP® HLA-C low resolution, resp. DQ-DR SSP Combi Tray (Olerup SSP AB, Sweden) (Olerup and Zetterquist 1992; Olerup et al. 1993). Amplification reactions were prepared according to manufacturers’ protocols, and electrophoresis was performed in a 1.5 % agarose gel for 20 min at 10 V/cm.

### Statistical analysis

Allele frequencies in the study group and in the controls were evaluated by direct counting. Data were tested for the goodness of fit between the observed and expected genotype frequencies, and no deviations from the Hardy–Weinberg equilibrium were found. Associations of particular alleles with psoriasis were expressed as odds ratios (OR) with 95 % confidence intervals (CI) calculated according to Woolf’s formula. Statistical significance of these associations was assessed using the *P* values defined by the two-sided Fisher’s exact test. As multiple comparisons were made, Bonferroni’s correction was applied multiplying the obtained *P* values by the number of alleles considered at each locus (*P*_cor_). The level of significance was set at *P*_cor_ < 0.01. All calculations were performed using the data analysis and graphing software OriginPro version 8.1.

## Results

The investigated HLA class I and class II allelic frequencies observed in 147 patients with PV and in the control group are given in Tables 2, 3 and 4. Differences between the cohorts were expressed by odds ratios and *P*_cor_ values. They are indicated in the tables only when statistically significant, i.e. when the *P* value corrected for the number of alleles considered at each locus was <0.01.

**Table 2:** Distribution of HLA-C alleles in patients with psoriasis and controls

HLA-C	Psoriasis vulgaris (2*n* = 294)		Controls (2*n* = 214)		OR	95 % CI	*P*_cor_
	Count	*f* (%)	Count	*f* (%)			
*01	13	4.42	13	6.07	0.71	0.32–1.59	n.s.
*02	11	3.74	12	5.61	0.66	0.28–1.52	n.s.
*03	24	8.16	19	8.88	0.92	0.49–1.72	n.s.
*04	42	14.29	34	15.89	0.88	0.54–1.45	n.s.
*05	10	3.40	9	4.21	0.80	0.32–2.00	n.s.
*06	69	23.47	16	7.48	3.85	2.13–6.67	<0.0001
*07	50	17.01	58	27.10	0.55	0.36–0.85	n.s.
*08	2	0.68	7	3.27	0.20	0.04–0.99	n.s.
*12	52	17.69	32	14.95	1.22	0.76–2.00	n.s.
*14	1	0.34	0	0	2.19	0.09–54.12	n.s.
*15	4	1.36	3	1.40	0.97	0.22–4.35	n.s.
*16	13	4.42	4	1.87	2.44	0.78–7.69	n.s.
*17	1	0.34	5	2.34	0.14	0.02–1.23	n.s.
*18	2	0.68	2	0.93	0.72	0.10–5.26	n.s.

*OR* odds ratio, *CI* confidence interval, *P*_*cor*_ corrected *P* value, *n*.*s*. not significant

**Table 3:** Distribution of HLA-DRB1 alleles in patients with psoriasis and controls

HLA-DRB1	Psoriasis vulgaris (2*n* = 294)		Controls (2*n* = 388)		OR	95 % CI	*P*_cor_
	Count	*f* (%)	Count	*f* (%)			
*01	11	3.74	42	10.82	0.32	0.16–0.64	<0.001
*03	20	6.80	31	7.99	0.84	0.47–1.52	n.s.
*04	26	8.84	35	9.02	0.98	0.57–1.67	n.s.
*07	85	28.91	54	13.93	2.56	1.72–3.70	<0.0001
*08	8	2.72	10	2.58	1.05	0.41–2.70	n.s.
*09	2	0.68	1	0.26	2.63	0.24–33.33	n.s.
*10	4	1.36	4	1.03	1.33	0.33–5.26	n.s.
*11	36	11.57	63	16.24	0.72	0.46–1.12	n.s.
*12	13	4.42	5	1.29	3.57	1.25–10.00	n.s.
*13	38	12.93	56	14.43	0.88	0.56–1.37	n.s.
*14	11	3.74	11	2.83	1.33	0.57–3.13	n.s.
*15	23	7.82	52	13.40	0.55	0.33–0.92	n.s.
*16	19	6.47	24	6.18	1.05	0.56–1.96	n.s.

*OR* odds ratio, *CI* confidence interval, *P*_*cor*_ corrected *P* value, *n*.*s*. not significant

**Table 4:** Distribution of HLA-DQB1alleles in patients with psoriasis and controls

HLA-DQB1	Psoriasis vulgaris		Control subjects		OR	95 % CI	*P*_cor_
	(2*n* = 294)		(2*n* = 388)				
	Count	*f* (%)	Count	*f* (%)			
*05	51	17.35	85	21.91	0.66	0.45–0.97	n.s.
*06	50	17.00	92	23.71	1.75	1.23–2.50	n.s.
*02	86	29.25	74	19.07	1.09	0.79–1.49	<0.01
*03	103	35.04	129	33.25	0.66	0.20–2.22	n.s.
*04	4	1.36	8	2.06	0.75	0.51–1.10	n.s.

*OR* odds ratio, *CI* confidence interval, *P*_*cor*_ corrected *P* value, *n*.*s*. not significant

As may be seen in Table 2, the strongest association among HLA-C alleles is conferred by the C*06 allele. The allelic frequency of C*06 was 23.5 % in patients compared to 7.5 % in controls, which is an extremely statistically significant difference (OR = 3.85, *P*_cor_ = 0.000025). Out of the HLA-DRB1 alleles presented in Table 3, only DRB1*07 was positively associated with the disease, showing a frequency of 28.9 % in the patients versus 13.9 % in the control group. This association is again extremely statistically significant (OR = 2.56, *P*_cor_ = 0.000019). On the contrary, the DRB1*01 allele was associated with PV negatively (OR = 0.32, *P*_cor_ = 0.0097). Out of the DQB1 alleles, the DQB1*02 was observed to have a significantly increased frequency in the patients (29.3 % versus 19.1 %, OR = 1.09, *P*_cor_ = 0.0095) as shown in Table 4.

We have divided our 147 patients into two subsets according to the presence (46.3 %) versus absence (53.7 %) of psoriatic arthritis. No statistically significant differences in the investigated HLA allele frequencies were observed between the two cohorts. On the contrary, significant differences were noted when each of the two subsets was compared with population controls, namely a strong positive association of HLA-C*06 with psoriasis with the presence of PsA (OR = 3.94, *P*_cor_ < 0.0001) and a slightly weaker association with psoriasis alone (OR = 3.72, *P*_cor_ < 0.0001). Similarly, there was a statistically significant association of HLA-DRB1*07 with psoriasis + PsA (OR = 2.75, *P*_cor_ < 0.0001) and a weaker association with psoriasis without PsA (OR = 2.27, *P*_cor_ = 0.002).

## Discussion

Although the aetiology of psoriasis vulgaris is still unknown, there is a common consensus that it is a complex disorder occuring in a sensitive genetic territory. Multiple genes seem to be involved in the pathogenesis of psoriasis; however, the major genetic determinant is located within the PSORS1 segment of the HLA region on chromosome 6p21.3, as reported by several independent groups (Nair et al. 2000; Martinez-Borra et al. 2003).

Our study confirmed the strong positive association of psoriasis with HLA-C*06, observed mainly in Caucasoid populations as well as in several other ethnically different groups. There are only a few studies from oriental populations, e.g. the Japanese reported association with other HLA-C alleles, most often with the HLA-C*07 allele (Roitberg-Tambur et al. 1994; Asahina et al. 1996). Interestingly, the occurrence rate of HLA-C*07 was increased also in our psoriatic patients, though only on the edge of the statistical significance (*P*_cor_ = 0.07).

The HLA-C*06 allelic frequency of 23.5 % observed in our psoriatic subjects (compared to 7.5 % in the controls) highly corresponds with that of 23.9 % observed in the PV patients of neighbouring Poland and fits into the general image seen in the Central European geographic region (Szczerkowska-Dobosz et al. 1996; Łuszczek et al. 2003).

Though HLA-C*06 is undoubtedly associated with PV, limited data exist to explain the functional role of HLA-C in the pathogenesis of psoriasis. According to many researchers, it is unlikely that HLA-C itself is the susceptibility gene (Elder et al. 2001; Orru et al. 2005; Pasic et al. 2009). HLA-C is most probably a marker of another closely linked gene, or a block of genes at a distinct locus. Thus, major effort has been put into the identification of candidate genes close to HLA-C such as the corneodesmosin gene, which encodes an adhesive protein expressed by keratinocytes or the alpha-helix coiled-coil rod homologue gene. However, there is a lack of evidence confirming their direct involvement in the development of psoriasis, and none of the candidates are convincingly associated with psoriasis independent of HLA-C (Tazi-Ahnini et al. 1999; O’Brien et al. 2001; Capon et al. 2002, 2003).

The HLA-C*06 allele is the only genetic variant repeatedly observed to be associated with the phenotypic features of PV, especially with an early onset of the disease as confirmed among others by Szczerkowska-Dobosz et al. (2004). According to Henseler and Christophers (1985), patients may be classified into two distinct types: type I (age at disease onset before 40 years, familial occurrence and high frequency of HLA-C*06) and type II psoriasis (age at onset after 40, occuring sporadically, characterized by a weak association with HLA-C*06). However, it proved difficult to classify our subjects as either having the type I or type II psoriasis as many patients were in an intersection of the two types showing features of both groups, and thus, we failed to confirm this observation. Comparison of the two types was also limited by the fact that we had only a relatively small fraction of late-onset patients (17.6 %).

In order to determine whether HLA-C*06 and, respectively, other alleles are associated with PsA itself or primarily with psoriasis, we have compared the allele frequencies obtained in psoriatic patients with PsA with frequencies in patients having psoriasis alone. Our results suggest that the primary association with HLA-C*06 and HLA-DRB1*07 is with psoriasis and not with PsA per se.

Significant overrepresentation of the neighbouring HLA class II alleles, namely the DRB1*07 and DQB1*02 in our study group, is most probably due to the fact that they are contained within the same extended haplotype. This particular risk haplotype was observed also in Croatian psoriasis patients and was named EH-57.2 (Kastelan et al. 2003).

Many authors have reported some alleles that occur in lower occurrence rates in patients with PV, indicating they may have a protective effect (Cassia et al. 2007; Kundakci et al. 2002; Łuszczek et al. 2003). The only allele with a significantly decreased frequency in our patients was the HLA-DRB1*01 (OR = 0.32, *P*_cor_ < 0.001). However, as the palette of published “protective” alleles is very variegated and they show only mild association, we assume that they occur at the expense of the highly overrepresented predisposing alleles.

In conclusion, our study provides the first report on HLA class I and class II allele association with psoriasis vulgaris in the Slovak population. Our data point toward the alleles HLA-C*06, DRB1*07 and DQB1*02, respectively, as major genetic risk factors for PV. Precise identification of genetic factors that increase the risk of psoriasis and psoriatic arthritis will help to elucidate the pathogenesis of this troubling disease and identify targets for a more specific and effective therapy.
